# Anastasis: recovery from the brink of cell death

**DOI:** 10.1098/rsos.180442

**Published:** 2018-09-19

**Authors:** Ho Man Tang, Ho Lam Tang

**Affiliations:** 1 Institute for Basic Biomedical Sciences, Johns Hopkins University School of Medicine, Baltimore, MD 21205, USA; 2 Department of Neurosurgery, Johns Hopkins University School of Medicine, Baltimore, MD 21205, USA; 3 School of Life Sciences, Chinese University of Hong Kong, Shatin, Hong Kong

**Keywords:** anastasis, apoptosis, mutagenesis, programmed cell death, reversal of apoptosis, reversal of cell death process

## Abstract

Anastasis is a natural cell recovery phenomenon that rescues cells from the brink of death. Programmed cell death such as apoptosis has been traditionally assumed to be an intrinsically irreversible cascade that commits cells to a rapid and massive demolition. Interestingly, recent studies have demonstrated recovery of dying cells even at the late stages generally considered immutable. Here, we examine the evidence for anastasis in cultured cells and in animals, review findings illuminating the potential mechanisms of action, discuss the challenges of studying anastasis and explore new strategies to uncover the function and regulation of anastasis, the identification of which has wide-ranging physiological, pathological and therapeutic implications.

## 1. Introduction

**Figure 1.**
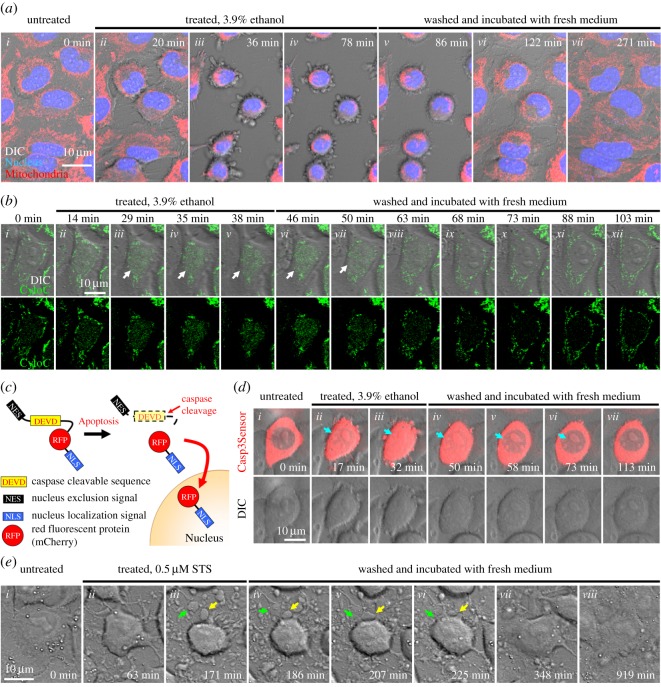
Cell recovery after attempted late apoptosis. (*a*) Time-lapse live cell confocal microscopy showing reversal of apoptosis in HeLa cells. To visualize mitochondria and nucleus, cells were stained with MitoTracker (red fluorescence) and Hoechst (blue fluorescence) for confocal microscopy, respectively. Cell morphology was observed with differential interference contrast (DIC) microscopy. In healthy cells, mitochondria formed a tubular network which extended throughout the cytoplasm (*i*). During exposure to a cell death stimulus of 3.9% ethanol, cells displayed morphological hallmarks of apoptosis including mitochondrial fragmentation, nuclear condensation, cell shrinkage, and plasma membrane blebbing (*ii–iv*). After washing and incubating the apoptotic dying cells with fresh culture medium, reversal of apoptosis occurred as indicated by morphological recovery of the cells (*v–vii*). The original movie can be viewed in electronic supplementary material. (*b*) Recovery of an apoptotic cell after mitochondrial cytochrome *c* release to cytosol. Time-lapse live cell confocal microscopy of a HeLa cell expressing a fusion protein of cytochrome *c*-GFP (Cyto*C*). Before cell death induction, cytochrome *c* localized in tubular mitochondria (*i*). During apoptosis induced by 3.9% ethanol, cytochrome *c* was released to cytosol (*ii*–*v*). After removal of the cell death inducer, cytosolic cytochrome *c* was reduced in the recovered cell (*vi*–*xii*). Merged images of cytochrome *c*-GFP (green fluorescence) and DIC for cell morphology (top row), and images of cytochrome *c*-GFP only (bottom row). White arrows indicate cytosolic signal of cytochrome *c*-GFP. (*c*) Schematic diagram of a caspase-3 biosensor fusion protein NES-DEVD-RFP-NLS (Casp3Sensor). (*d*) Recovery of an apoptotic cell after caspase-3 activation. Time-lapse live cell confocal microscopy of a HeLa cell expressing fusion protein of Casp3Sensor. In the healthy cell, the Casp3Sensor localized in cytosol (*i*). During apoptotic induction of 3.9% ethanol, the caspase-3 cleaved biosensor translocated from cytosol to nucleus (*ii*,*iii*). After removal of the death stimulus, the signal of nuclear Casp3Sensor was diminished in the recovered cell (*iv*–*vii*). Merged images of Casp3Sensor (red fluorescence) and DIC for cell morphology (top row), and images of DIC only (bottom row). Blue arrows indicate nuclear signal of Casp3Sensor. (*e*) Apoptotic bodies fuse to cell body during cell recovery. Time-lapse live cell DIC microscopy of a healthy HeLa cell (*i*), the same cell after treating with a cell death stimulus of 0.5 µM staurosporine (STS; *ii*,*iii*), and then being washed and further incubated with fresh culture medium to remove the staurosporine (*iv–viii*). Green and yellow arrows indicate two apoptotic bodies that fused to a cell body during recovery of the cell.

Programmed cell death is an essential component of life. Among over 20 forms of programmed cell death that have been proposed [1–3], apoptosis is by far the most well studied for its regulatory mechanisms in cell suicide, and essential roles in embryonic development and normal homeostasis by eliminating the unwanted, injured or dangerous cells in the body [4–6]. While genetic or pharmaceutical manipulation can allow dying cells to survive that otherwise would normally die [7–9], initiation of apoptosis is generally believed to represent an irreversible commitment to cell death [10,11]. Events canonically marking this ‘point of no return’ in apoptosis include release of mitochondrial cytochrome *c* into the cytosol [12–17], activation of execution caspase proteases [18–20], and their associated or downstream events [1], such as DNA damage, cell surface exposure of ‘eat me’ signals, cell shrinkage and fragmentation of the cell body. However, a growing body of evidence has demonstrated that dying cells can exit the initiated death process and recover, even at late stages generally accepted as beyond the ‘point of no return’. We coined a term *anastasis* (*Αναστ*ά*ση*ς), which means ‘rising to life’ in Greek [21], to describe the recovery of dying cells after brink of death, using reversal of apoptosis as the first example. More recent studies expand the anastasis field that dying cells can recover following important cell death hallmarks of apoptosis and potentially other forms of cell death process (figure 1 and table 1). Removal of the death stimulus is sufficient to allow anastasis to occur *in vitro* and *in vivo*, indicating that anastasis is an intrinsic recovery phenomenon. In this review, we gather and summarize the strength of evidence supporting anastasis, discuss the possible mechanisms of anastasis at each step of the cell death process and address the key challenges of studying and furthering our understanding of anastasis. Given the novelty of this field, we also look forward to some of the potential physiological, pathological and therapeutic implications of anastasis, and propose future directions for the study of anastasis.

**Table 1.** Reversal of cell death events.

cell death events	references
externalization of phosphatidylserine (*in vitro*)	[21–24]
externalization of phosphatidylserine (*in vivo*)	[25]
cytochrome *c* release	[26]
incomplete MOMP	[27]
mitochondrial fragmentation	[21,26,28,29]
caspase activation (*in vitro*)	[21,26,28–33]
caspase activation (*in vivo*)	[34,35]
plasma membrane blebbing	[21,26,28,29]
cell shrinkage	[21,24,26,28,29,31,32]
DNA damage	[21,27,30]
nuclear condensation	[21,26,28,29,32]
RIPK3 activation	[24,36]
apoptotic body formation and cell fragmentation	[26]

## 2. Cell death process: considered irreversible

Apoptosis is executed by the sophisticated cellular demolition mechanisms, in which mitochondrial cytochrome *c* release and caspase activation are critical steps in this process of cell suicide [12–20]. During apoptosis, pro-apoptotic cell death factors translocate to and fragment mitochondria, leading to mitochondrial outer membrane permeabilization (MOMP), which releases apoptogenic factors into the cytosol [14,37–39]. These factors include cytochrome *c* to initiate the caspase protease cascade [40,41], Smac/DIABLO to suppress the inhibitor of apoptosis protein (IAP) for enhancing caspase activation [42,43], and specific DNases for apoptosis such as apoptosis-inducing factor (AIF) and endonuclease G (EndoG), which enzymatically cleave the genome [44–46]. Activated caspases mediate apoptosis by directly and indirectly cleaving hundreds of cellular substrates. For example, caspases activate DNA fragmentation factor/caspase-activated DNase (DFF40/CAD) that destroys the genome by cleaving its inhibitor, DFF45/ICAD [47,48], and cleave DNA-repairing enzyme Poly(ADP)-ribose polymerase-1 (PARP) that plays a critical role in maintaining genomic stability [49,50]. Activated caspases also cleave flippases at the plasma membrane, leading to cell surface exposure of phosphatidylserine, which then acts as an ‘eat me’ signal recognized by phagocytic cells [51]. Caspase cleavage of cytoskeletons and their regulators contributes to plasma membrane blebbing, cell shrinkage and fragmentation [52–60], signalling and facilitating the phagocytosis of apoptotic cells and recycling of their contents [4,61,62].

Importantly, apoptosis is a rapid and massive cellular destruction process [63]. The process to activate apoptosis is multivariate, requiring minutes to days or even longer after a death stimulus is applied. Once initiated, pro-apoptotic cell death factors such as BAX translocate to and fragment mitochondria within 15 min [64,65], leading to mitochondrial damage and release of apoptogenic factors including cytochrome *c* and SMAC to occur within 1–5 min [66–68], followed by rapid caspase activation and morphological features of apoptosis, including nuclear condensation, plasma membrane blebbing and cell shrinkage within 10–15 min [69–71]. While activated caspases execute cellular destruction by proteolysis of functional and structural components, apoptotic events also render mitochondria dysfunctional, disrupting cellular bioenergetics and metabolism [72–74]. Notably, mitochondrial damage or caspase activation alone is sufficient to cause cell death independently [18,39]. Therefore, apoptosis is generally considered to be irrevocable [10,11], especially at late times after these critical cell death-executing activities occur. However, recent studies reveal that recovery of dying cells is possible, even after reaching these critical cell death events.

## 3. Evidence and potential mechanisms of anastasis

**Figure 2.**
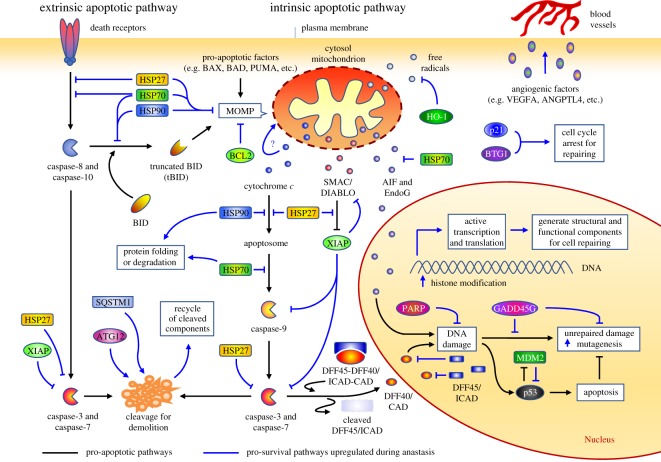
Proposed mechanism of anastasis during cell recovery. Upregulation of pro-survival pathways identified during anastasis interact with the apoptosis network to suppress initiated death cascade and promote cell recovery.

Can a dying cell recover from the brink of cell death after reaching the generally assumed ‘points of no return’? If so, how can a dying cell reverse a cell death decision? Recovery should involve arresting programmed death cascades, restoring normal cellular functions and repairing damage. While the precise mechanisms remain unclear, recent studies have demonstrated anastasis and provided new insights into the potential strategies possibly adopted by anastatic cells to halt and reverse the initiated cell death process (figure 2).

### 3.1. Recovery after cytochrome *c* release

Considering that dysfunctional energy production of damaged mitochondria, initiation of the proteolytic caspase cascade and demolition of the genome can each independently cause cell death, recovery of dying cells after cytochrome *c* release by MOMP would seem unlikely [12–17]. Nevertheless, recent studies reveal an unexpected reversibility of apoptosis after mitochondrial fragmentation (figure 1*a*) and cytochrome *c* release (figure 1*b*). For example, single cell live microscopy study demonstrated that cytochrome *c*-releasing and mitochondria-fragmented dying cells are able to recover [26], even after plasma membrane blebbing, a downstream hallmark of effector caspase activation [57,58], is observed [26]. This suggests a reversibility of apoptosis even after these once considered irrevocable steps. Besides, recovery of dying cells after cytochrome *c* release could also occur *in vivo*. During heart failure, significant numbers of dying cardiomyocytes exhibit several hallmarks of apoptosis, including mitochondrial release of cytochrome *c* and activation of caspase-3, but these cells maintain normal nuclear morphology with the absence of terminal morphological features of apoptosis, indicating that the dying cells may not complete the apoptotic process [75–77]. This phenomenon, termed *apoptosis interruptus*, suggests possible arrest or interference with the apoptotic process [78], and therefore, could explain the clinical observation on recovery of cardiomyocytes during reversal of heart failure, in which the failing heart was unloaded with a left ventricular assist device to reduce the physical stress on the heart that can cause apoptosis [79,80].

Mitochondria serve as the powerhouses of the cell [14,81]. Release of cytochrome *c* from mitochondria to cytosol causes mitochondrial damage and dysfunction during apoptosis [14,82]. How can anastatic cells obtain energy to recover from apoptosis? By contrast to what was originally interpreted as an ‘all or nothing’ event [12,66,67], incomplete release of cytochrome *c* is observed in a subset of mitochondria in dying cells [26,83,84], possibly due to incomplete mitochondrial outer membrane permeabilization (iMOMP) [27]. This suggests that some remaining intact or partially functional mitochondria may be available to support early energy requirements of anastasis. At later times in anastasis, mitochondria show recovery, as determined by time-lapse live cell microscopy revealing fragmented mitochondria can fuse and regain their normal tubular structure (figure 1*a*) [21,26,28,29,85], thereby restoring the powerhouse to provide bioenergetics and metabolic support during subsequent recovery. Of note, some cell types, such as neurons and cardiomyocytes, are able to resist cell death induction when cytochrome *c* is introduced into the cytosol by microinjection [86,87], suggesting the existence of intrinsic mechanisms for handling the cytosolic cytochrome *c* without triggering inevitable cell death.

Logically, the recovering cells will need to inhibit cytochrome *c* release, degrade cytosolic apoptogenic factors and remove the damaged mitochondria to restore energy production, but the related molecular mechanism remains unclear. A recent time-course whole-genome gene expression microarray study provides mechanistic insights by identifying the molecular signature of anastasis in primary mouse liver cells, which reveals striking temporal changes of gene transcription during anastasis [29]. This signature included upregulation of the autophagy-related protein Atg12, autophagic adaptor protein Sqstm1, heat shock proteins Hspb1, Dnajb1, HSPa1a and Hsp90aa1 (HSP27, HSP40, HSP70 and HSP90, respectively, for human homologue), and Hmox1 for encoding for heme oxygenase (HO-1) [29]. ATG12 and SQSTM1 are key regulators of mitochondrial homeostasis and remove impaired mitochondria by mitophagy [88–91], suggesting that autophagy may be involved in degrading damaged mitochondria after cytochrome *c* release in anastatic cells. ATG12 also promotes the rapid degradation of cytosolic cytochrome *c* in the absence of caspase activation [92,93]. Besides, the chaperone proteins HSP27 and HSP70 have been shown to suppress the mitochondrial release of cytochrome *c* [94–98], and cooperate with HSP90 to inhibit cytochrome *c*-mediated caspase activation [99–105], thereby halting further caspase activation. Studies also demonstrated that HSP70 and HSP40 suppress mitochondrial translocation of pro-apoptotic BAX and caspase-8-mediated activation of pro-apoptotic BCL-2 family member BID [106,107], while HSP27 suppresses mitochondrial translocation of the cleaved BID [94], thereby preventing further MOMP-mediated leakage of apoptogenic factors. HSP70 also interacts with and suppresses AIF and EndoG, preventing DNA destruction after MOMP [108–110]. Expression of heme oxygenase could also promote cell survival by removing free radicals that are generated during apoptosis [111]. Collectively, the upregulation of this set of genes during anastasis suggests candidate regulators that degrade released cytochrome *c*, suppress the activated executioners and restore a functional network of healthy mitochondria that is essential for energy production.

### 3.2. Arrest of activated caspase cascade

During the execution phase of apoptosis, activated caspases carry out the proteolytic destruction of key functional and structural components of the cell [18,20]. This rapid and massive cellular demolition is initiated and amplified through multiple apoptotic pathways mediated by caspases (figure 2). Activation of caspases ultimately leads to fragmentation of organelles [18,20,38], destruction of the genome by activating apoptotic DNases [47,48], cell surface exposure of phagocytic signals [51] and formation of apoptotic bodies in preparation for the phagocytic clearance of dead cells [4,61,62]. Activated caspases also cleave BID, which subsequently translocates to mitochondria to further promote MOMP to accelerate the apoptotic cascade [112–116]. Given the critical role of caspases in initiating and amplifying death signals that destroy or cripple a multitude of cellular processes, apoptosis used to be considered to be inevitable after caspase activation [18–20].

Interestingly, recent studies demonstrate that reversal of apoptosis can actually occur after caspase activation in cultured cells and in live animals. Time-lapse live cell microscopy of human cervical cancer HeLa cells expressing a caspase-3 biosensor detected caspase activation after ethanol-induced apoptosis, and showed that the same cells were able to recover and eliminate the cleavage-activated biosensor after being washed and incubated with fresh medium (figure 1*c,d*) [21,26]. These caspase-activated cells can also recover after mitochondrial fragmentation, cytosolic and nuclear condensation, plasma membrane blebbing and cell shrinkage [21,26,85], thereby indicating that apoptosis can be reversible at the cell execution stage.

It is technically challenging to demonstrate anastasis in animals, because cells that underwent anastasis can be morphologically indistinguishable from surrounding healthy cells. To overcome this difficulty, an *in vivo* CaspaseTracker biosensor was developed [34], which can be activated by effector caspase to trigger permanent fluorescent protein expression within anastatic cells and their progeny, allowing the tracking of cell fate [34]. Studies done *in vivo* demonstrate that after transiently exposing *Drosophila melanogaster* transgenic for CaspaseTracker biosensor to environmental insults, such as protein starvation or cold shock that trigger apoptosis in various tissues including egg chambers (somatic and germ cells), fluorescent protein was permanently expressed in these cells of the recovered flies, but not in those of untreated control flies [34,85]. This indicates that anastasis can occur after caspase activation in animals. Notably, this biosensor is also activated both during and after development [34,35,85], suggesting a potential involvement of anastasis during embryogenesis and normal homeostasis.

Broadening studies of non-apoptotic caspase activity reveal the existence of a still poorly understood strategy for cells to handle the activated caspases without the cells being killed. These include regulation of neuronal activity [117–122], spermatid individualization [123,124], microRNA processing [125], cell proliferation [126] and cell fate patterning [127]. Remarkably, cultured cells have been shown to tolerate sublethal caspase activity without triggering apoptosis [30,128–130]. These studies indicate that caspase activation is not the ‘point of no return’. Although it is still unclear whether cells use the same or different mechanism to manage the activated caspases for anastasis and non-apoptotic caspase activity, several genes that are specifically upregulated during reversal of apoptosis could serve as suppressors of the caspases [29]. For example, HSP27 has been shown to bind procaspase-3 and inhibit the caspase-9-mediated proteolysis required for the activation of caspase-3 [100], while HSP27, HSP70 and HSP90 can suppress apoptosome formation and activation of caspase-9 [99–105], thus preventing further activation and amplification of the caspase cascade. Upregulation of murine double minute (MDM2), an inhibitor of p53 [131–133], occurs in anastatic cells [29], and that could suppress the p53-mediated pro-apoptotic DNA damage response during reversal of apoptosis. Expression of MDM2 can also activate the X-linked inhibitor of apoptosis protein (XIAP) [134], which arrests the death cascade by inhibiting the activated initiator and executioner caspases [135–140], and promoting the degradation of Smac/DIABLO [141,142]. Transcription is also active during anastasis [21,29,31], and that can generate building blocks for the recovering cells to repair damage and regain normal morphology. Interestingly, anastatic cells also display upregulation of potent angiogenic factors, such as ANGPTL4 and VEGFA [29], which promote angiogenesis and vascular permeability [143–146]. This could enhance anastasis by facilitating the nutrient supply and removal of cellular wastes such as those generated by degradation of the caspase-cleaved products, but this has not yet been confirmed experimentally.

### 3.3. Repairing DNA damage

DNA damage is a hallmark of apoptosis, executed by apoptotic DNases such as AIF and EndoG being released from the mitochondria [44–46], and DFF40/CAD activated by caspases that cleave its inhibitor of DFF45/ICAD [47,48]. Executioner caspases also abolish the DNA repair system in dying cells by cleaving the DNA-repair enzyme PARP [49,50].

Anastasis can occur at this late stage in the cell death process. Mouse primary liver cells and non-cancerous NIH3T3 fibroblasts that had undergone apoptosis following exposure to ethanol, a cell death inducer, and showed mitochondrial release of AIF and EndoG, cleavage of ICAD and PARP and breakage of DNA strands, were able to reverse apoptosis after removing the death inducer and restoring normal culture conditions [21]. Although recovery is possible, some surviving cells acquired new chromosomal abnormalities and underwent an oncogenic transformation, as indicated by loss of contact inhibition of growth (focus formation) and anchorage-independent growth (proliferation in soft agar) [21]. An increased frequency of cells with micronuclei, a biomarker of DNA damage [11,147], has also been observed in both primary cells and cancer cell lines that recovered from apoptosis [21,29].

DNA damage and oncogenic transformation can also be promoted by iMOMP and sublethal activation of caspase-3, presumably through apoptotic DNases, whereas suppressing caspase activation or apoptotic DNase activity has the opposite effect of inhibiting transformation [28,30]. Studies with leukaemia cell lines also showed that sublethal apoptosis induction promotes MLL gene translocations, TEL breaks and formation of TEL-AML1 fusion [148–150], which are often detected during cancer progression [151,152]. These observations support the notion that activation of apoptotic machines can promote mutagenesis by damaging the genome when the cells attempt apoptosis but do not die, and then recover [21]. Therefore, it is possible that DNA damage sustained during apoptosis may not be appropriately repaired during anastasis, leading to the acquisition of new mutations.

How can dying cells with damaged DNA survive through anastasis? One possibility is that during anastasis, upregulation of the ubiquitin ligase MDM2 occurs [29], leading to degradation of the tumour suppressor p53, thereby suppressing the pro-apoptotic p53-mediated DNA damage response [131–133]. HSP70 upregulated during anastasis may also interact with and suppress AIF and EndoG to stop DNA destruction initiated after MOMP [29,108–110]. While being cleaved by caspases during apoptosis, the expression of ICAD and PARP has been shown to return to pre-apoptosis levels during anastasis [21], enabling ICAD to inhibit the DNase activity of CAD, and PARP to repair DNA damage in anastatic cells. In addition, cell cycle arrest is important for DNA repair [153,154], and upregulation of the cell cycle arrest genes such as Btg1, Cdkn1a and Trp53inp1 occurs during anastasis [29].

### 3.4. Reunion after formation of apoptotic bodies

Remarkably, anastasis can still occur even after the fragmentation of the dying cells [26,85]. Time-lapse live cell microscopy has demonstrated that HeLa cell fragments resulting from the formation of apoptotic bodies can coalesce to an apparently normal morphology after removing the cell death inducer staurosporine (figure 1*e*) [85]. A similar coalescence of fragmented cells has also been observed with the cultured human small cell lung carcinoma H446 cell line after recovery from ethanol-induced apoptosis [26]. As mentioned above, recovered cells often display an increased number of micronuclei and chromosomal abnormalities [21,26,29], suggesting unrepaired DNA damage after anastasis. Failure of apoptotic bodies with broken chromosomes to faithfully reassociate during anastasis may lead to increased aneuploidy [21,26].

It is unclear how the pieces of fragmented apoptotic cells can coalesce back to a seemingly normal structure. Recent studies have revealed that surface exposure of phosphatidylserine may be a key requirement for different types of cell fusion, including macrophage polykaryon formation [155], myotube formation [156,157], viral infection [158] and fusion of severed axons [159]. This leads to speculation that the fragmented cells with externalization of PS could use this strategy for fusion and repair during anastasis.

Under normal physiological conditions, apoptotic body formation is followed by phagocytosis. Perhaps related to recovery after the formation of apoptotic bodies may be the intriguing question of whether engulfed cell fragments can undergo anastasis. Internalized living cells undergoing entosis have been shown to escape their cannibalized state, ultimately surviving and proliferating [160]. However, entosis is not typical phagocytosis and is distinguished as an invasion of a living cell into the cytoplasm of another cell [160], and so it may not represent how macrophages engulf apoptotic cell fragments [4,38].

### 3.5. Removal of externalized phosphatidylserine

Under normal conditions, phosphatidylserine (PS) is restricted to the inner leaflet of the plasma membrane. During apoptosis, however, PS becomes exposed on the outer surface of the cell and represents an ‘eat me’ signal that facilitates rapid engulfment by phagocytes [61,62]. Despite the deployment of surface PS, dying cells can undergo anastasis. For example, apoptotic BCL_1_.3B3 B lymphoma cells (induced by pro-apoptotic anti-immunoglobulin antibodies [22]) or mouse mammary carcinoma MOD cells (with temperature-sensitive p53 and induced by shift to permissive temperature [23]) were able to proliferate after removing the death inducer, indicating that anastasis can occur after surface exposure of PS [22,23]. Anastasis after the PS exposure has also been observed in neonatal rat primary cardiomyocytes and the mouse cardiac muscle HL1 cell line after transient cell death induction of ethanol [21,26].

It is expected that anastatic cells need to remove this ‘eat me’ signal in order to escape phagocytosis, and there is evidence that this can happen. Recovering cells have been shown to retain surface-exposed PS for only a few hours, suggesting that the removal of previously exposed PS is an active process during anastasis [21,26], although the exact molecular mechanism is unclear. Recovery after PS exposure has also been observed in live animals. PS identified by annexin V in rabbit and mouse cardiomyocytes recovering from apoptosis induced by transient ischaemic injury, was detected inside the surviving cells [25]. This suggests the internalization of PS in the anastatic cells to its pre-apoptotic plasma membrane location, rather than, for example, engulfment of dead cells by their neighbouring cells, but this interpretation requires further evaluation and corroboration.

## 4. Remaining key questions and challenges

Anastasis is a new field, and the current gaps in our knowledge raise fundamental questions about which kinds of cell death processes are reversible, what strategies can be developed to overcome the challenges of identifying the functions and regulatory pathways of anastasis, and how knowledge of anastasis might be translated to pharmacological intervention.

Anastasis is a term coined to describe the cell recovery phenomenon that reversal of apoptosis has been reported as the first example [21]. Interestingly, recent subsequent studies suggest that other forms of cell death may also be reversible. Apart from apoptosis, the cell death stimulus staurosporine induces necroptosis [161,162], starvation triggers autophagy [163,164] and cold shock leads to necrotic injury characterized by plasma membrane rupture [34,165–167], and interestingly recovery of these dying cells has been observed *in vitro* or *in vivo* after being returned to optimal culture conditions [28,34,85]. In addition, and although genetic manipulation is required, RIPK3-activable biosensor-induced necroptotic cells have been shown to recover when the biosensor was turned off [24], further supporting that necroptosis is reversible. Research extending the study of anastasis to different forms of cell death can be expected to provide a deeper understanding of its functions, mechanisms and consequences.

Two major challenges complicate studying the reversibility of cell death processes. First, a single cell death inducer can activate multiple cell death pathways, in which interconnections and interactions allow for complicating ‘cross talk’ among them [161–168], making it difficult to test the reversibility of one specific pathway. To solve this problem, suppression of key regulators of specific cell death pathways, such as caspases for apoptosis, MLKL for necroptosis and LC3 for autophagy, will be required to isolate pathways and determine which kinds of cell death are reversible after induction by ‘broad-spectrum’ death conditions. Second, it remains technically challenging to detect and track anastasis, especially in animals where histologically recognizable markers of apoptosis disappear after anastasis, and there is not yet a specific biomarker identified for anastasis. The recent development of the CaspaseTracker biosensor provided a novel strategy to identify apoptotic dying cells and track the fate of anastatic cells in live animals [34,35,85], but is unable to unambiguously distinguish between apoptotic and non-apoptotic caspase activation [161–167,169], making the biosensor not necessarily apoptosis-specific. Development of a trackable system to specifically follow anastatic cells recovered after different kinds of cell death will facilitate these *in vivo* studies.

Developing and refining pharmacological agents and other strategies to modulate anastasis requires a solid understanding of its molecular mechanism. This is an area of ongoing investigation. Whole-genome gene expression studies have been performed on anastasis in mouse primary liver cells [29] and human cervical cancer HeLa cells [31] after ethanol-induced apoptosis, as well as in mouse NIH3T3 fibroblasts after RIPK3-activated biosensor-induced necroptosis [36], but the putative master regulators of the process remain elusive. It is possible that anastasis is mediated by multiple pathways rather than by a specific master regulator. Future studies of post-translational and epigenetic influences on anastasis will provide new insights into its regulation.

The existence of a definite irreversible checkpoint for cell death is also not known. Dying cells undergoing fragmentation and membrane permeabilization are generally expected to commit demise, but as we have discussed, some cells can still recover [26,34,85], suggesting that there is a new tipping point. While the mechanism is not known, it is possible that cell-specific thresholds exist for survival; for example, cytosolic microinjection of cytochrome *c* induces death in the neonatal rat dermal fibroblasts but not the rat cardiomyocytes [87]. Some cells, for example neurons, can also avoid initiation of the death cascade or live with activated cell executioners such as caspases [34], although this is not well understood [119,170]. Clearly, future work is required to fully define the checkpoint of cell death and anastasis.

## 5. Looking forward

**Figure 3.**
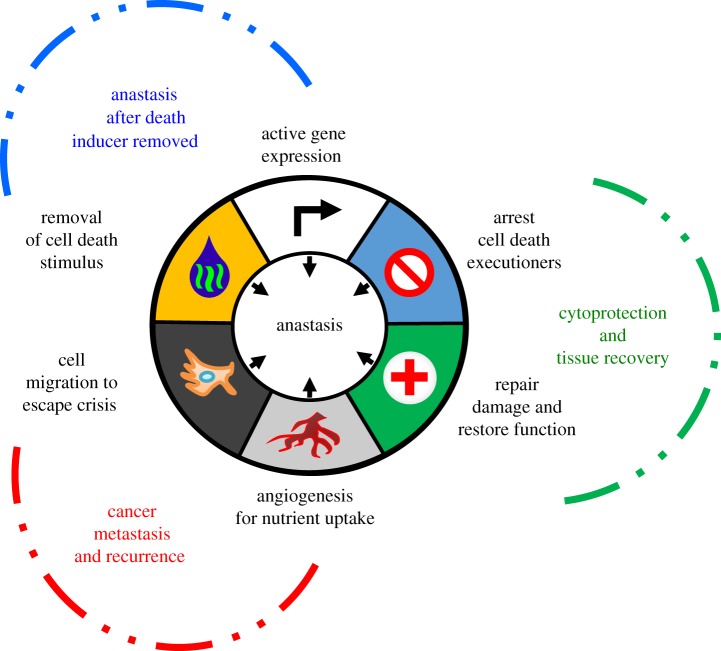
Emerging hallmarks of anastasis. This illustration encompasses the proposed features and consequences of anastasis.

There are numerous gaps in our current knowledge of anastasis. Here, we discuss the potential biological significance of this process (figure 3), though we must stress that further research is needed in the area. Anastasis may serve as a pro-survival force to balance apoptosis, and may therefore participate in the regulation of cell death and survival during embryonic development and normal homeostasis, as well as other physiological and pathological conditions.

Promoting anastasis may represent as a previously unrecognized beneficial mechanism of preserving differentiated cells that are difficult to replace, such as neurons and cardiomyocytes. For example, in flies, rats and rabbits, photoreceptor cells die if they are exposed to constant light, but this retinal degeneration can be reversed within the first few days by returning the animals to normal light–dark cycles [171–173]. As the proliferation of photoreceptor cells is not expected in the retina, anastasis may protect the retinal function by aiding the survival of severely injured photoreceptor cells, thus providing a novel approach to regenerative medicine. As another example, following brain injury neuronal cell death is mediated by cell death programmes that include apoptosis, as evidenced by caspase-3 and calpain activation [174]. Treatment of such traumas with anti-apoptotic erythropoietin and therapeutic hypothermia can reduce the extent of damage and improve functional outcomes [175,176], identifying apoptosis as a therapeutic target and raising the possibility that promoting anastasis might further enhance recovery from brain injury by rescuing dying neurons. As a third example, apoptosis occurring in cardiomyocytes of failing hearts [177,178], appears possible to arrest or reverse well beyond the expected ‘point of no return,’ as discussed above. It has been proposed that damaged heart tissue may undergo repair by phagocytic removal of the apoptotic cells and replacement by therapeutically induced compensatory division of healthy cardiomyocytes [179–181]. Anastasis may have a role in this process by limiting permanent damage and promoting cell survival following transient cell death-inducing stresses to the heart [78]. Anastasis can also occur in cultured primary liver cells [21,29], suggesting that it may augment recovery after liver injury as an additional or alternative mechanism other than cell division for liver regeneration [182].

Inhibiting anastasis may also have beneficial application. For example, cancer cells may employ anastasis as an escape tactic to survive cell death-inducing anti-cancer therapy, possibly contributing to cancer recurrence. Chemotherapy and radiotherapy kill cancer cells by inducing various types of cell death including apoptosis [183,184]. Primary cancers often exhibit dramatic initial responses to such therapies [185–189]. However, most metastatic cancers, including lung, brain, skin and pancreatic cancers, inevitably recur, leading to treatment failure [185–189]. Anastasis has been observed in various cultured human cancer cell lines including cervical cancer, small cell lung carcinoma, neuroblastoma, skin cancer, testicular cancer, liver cancer, breast cancer and prostate cancer [21,26,28,29,31–33,85], thereby suggesting that this process could be a common occurrence in cancers. Furthermore, upregulation of genes involved in cell migration (MMP9, MMP10 and MMP13) and angiogenesis (ANGPTL4, ANGPT2 and VEGFA) during anastasis [29] suggests a plausible connection between anastasis and cancer metastasis during a recurrence. More work is needed to determine whether this mechanism could contribute to cancer recurrence and spread, and whether targeting anastasis could provide a new strategy to fight cancer by suppressing the ability of apoptotic cancer cells to escape anti-cancer therapy.

As mentioned, some anastatic cells acquire permanent genetic changes or undergo an oncogenic transformation at a higher frequency than control cells that did not attempt cell death [21,26,29]. Therefore, anastasis may contribute to tumourigenesis by rescuing genetically damaged cells, potentially accounting for the observation that repeated tissue injury increases cancer risk. This has been observed in various tissues, such as alcohol-damaged liver [190,191] and chronic thermal esophageal injury caused by swallowing very hot beverages [192–194]. Moreover, anti-cancer therapy can trigger apoptosis in normal cells due to off-target effects, raising the possibility that anastasis to spare those cells following anti-cancer therapy could give rise to secondary cancers. For example, anastasis in haematopoietic stem cells could underlie the appearance of acute myeloid leukaemia arising after completion of anti-cancer treatments [195–197]. In that vein, anastasis occurring between cycles of genotoxic anti-cancer therapy could allow treatment-induced mutations to be perpetuated, leading to progression and evolution of drug resistance in recurrent cancers [185–189,198]. Thus, blocking or interfering with anastasis during anti-cancer therapy may offer a novel therapeutic strategy for preventing or arresting the progression of cancer and development of anti-cancer drug resistance.

In bacteria, yeast and plants, stress-induced mutagenesis is proposed to serve as an adaptive mechanism to cope with environmental changes by introducing new mutations for natural selection [199–202]. While stress-induced mutagenesis could benefit microorganisms and plants for the fitness of survival, it could be an unrecognized mechanism to cause genetic diseases when anastasis occurs in DNA-damaged germ cells. For example, epidemiological studies reveal that individuals born at the time of famines have a higher chance of developing transgenerational inheritable diseases such as breast cancer, coronary heart diseases, diabetes and obesity [203–210]. In response to environmental stresses such as starvation or temperature shock, germ cells undergo apoptosis, but fertility can resume shortly after the stressed animals are returned to a more normal environmental condition, presumably by restoring germ cell production [211–213]. Interestingly, recent studies demonstrated the occurrence of anastasis in germ cells of *Drosophila* after transient exposure to physiological and environmental stresses such as starvation or cold shock [34], thereby raising an intriguing possibility that recovered germ cells might be able to acquire new mutations from unrepaired DNA damage generated during apoptosis, leading to genetic alterations in their progeny [21,34]. If true, this mutagenetic mechanism might promote deleterious disease by causing changes along beneficial survival enhancing genetic alterations for natural selection, which could promote evolution especially in response to environmental changes.

## 6. Conclusion

The links between anastasis and tissue recovery, evolution of diseases and cell death decision remain to be elucidated. Further work is needed to determine whether anastasis is involved in these processes. Should they turn out to be true, studies on the physiological and pathological roles of anastasis could provide new insights into multidisciplinary fields of research that enhance our understanding in the control of cell death and survival, and also offer potential to identify new therapeutic approaches for cancers, heart failure, degeneration, tissue injury and regeneration medicine by mediating reversibility of cell death processes.

## Supplementary Material

Time-lapse movie showing reversal of apoptosis in HeLa cells.

## Data accessibility

Data are available from the cited references and the electronic supplementary material.
