# Correction to: ‘Anastasis: recovery from the brink of cell death’

**DOI:** 10.1098/rsos.181629

**Published:** 2018-10-10

**Authors:** Ho Man Tang, Ho Lam Tang

*R. Soc. open sci.* **5**, 180442. (Published 19 September 2018). (doi:10.1098/rsos.180442)

This correction refers to a production error that occurred in the citation of references 27–28 and 30–33.

**Table 1.** Reversal of cell death events.

cell death events	references
externalization of phosphatidylserine (*in vitro*)	[21–24]
externalization of phosphatidylserine (*in vivo*)	[25]
cytochrome *c* release	[26]
incomplete MOMP	[27]
mitochondrial fragmentation	[21,26,28,29]
caspase activation (*in vitro*)	[21,26,28–33]
caspase activation (*in vivo*)	[34,35]
plasma membrane blebbing	[21,26,28,29]
cell shrinkage	[21,24,26,28–30,32]
DNA damage	[21,27,31]
nuclear condensation	[21,26,28,29,32]
RIPK3 activation	[24,36]
apoptotic body formation and cell fragmentation	[26]

In Section 3.2, fourth paragraph, third sentence should read: ‘Remarkably, cultured cells have been shown to tolerate sublethal caspase activity without triggering apoptosis [31,128–130].’

In Section 3.2, fourth paragraph, third last sentence should read: ‘Transcription is also active during anastasis [21,29,32], and that can generate building blocks for the recovering cells to repair damage and regain normal morphology.’

In Section 3.3, third paragraph, first sentence should read: ‘DNA damage and oncogenic transformation can also be promoted by iMOMP and sublethal activation of caspase-3, presumably through apoptotic DNases, whereas suppressing caspase activation or apoptotic DNase activity has the opposite effect of inhibiting transformation [27,31].’

In Section 4, fourth paragraph, third sentence should read: ‘Whole-genome gene expression studies have been performed on anastasis in mouse primary liver cells [29] and human cervical cancer HeLa cells [32] after ethanol-induced apoptosis, as well as in mouse NIH3T3 fibroblasts after RIPK3-activated biosensor-induced necroptosis [36], but the putative master regulators of the process remain elusive.’

In Section 5, third paragraph, sixth sentence should read: ‘Anastasis has been observed in various cultured human cancer cell lines including cervical cancer, small cell lung carcinoma, neuroblastoma, skin cancer, testicular cancer, liver cancer, breast cancer and prostate cancer [21,26,28–30,32,33,85], thereby suggesting that this process could be a common occurrence in cancers.’

